# Classifying patient and professional voice in social media health posts

**DOI:** 10.1186/s12911-021-01577-9

**Published:** 2021-08-18

**Authors:** Beatrice Alex, Donald Whyte, Daniel Duma, Roma English Owen, Elizabeth A. L. Fairley

**Affiliations:** Talking Medicines Limited (SC447227), 25 Blythswood Square, Glasgow, G2 4BL Scotland UK

**Keywords:** Patient voice, Professional voice, Social media, Classification, Reddit, Twitter

## Abstract

**Background:**

Patient-based analysis of social media is a growing research field with the aim of delivering precision medicine but it requires accurate classification of posts relating to patients’ experiences. We motivate the need for this type of classification as a pre-processing step for further analysis of social media data in the context of related work in this area. In this paper we present experiments for a three-way document classification by patient voice, professional voice or other. We present results for a convolutional neural network classifier trained on English data from two different data sources (Reddit and Twitter) and two domains (cardiovascular and skin diseases).

**Results:**

We found that document classification by patient voice, professional voice or other can be done consistently manually (0.92 accuracy). Annotators agreed roughly equally for each domain (cardiovascular and skin) but they agreed more when annotating Reddit posts compared to Twitter posts. Best classification performance was obtained when training two separate classifiers for each data source, one for Reddit and one for Twitter posts, when evaluating on in-source test data for both test sets combined with an overall accuracy of 0.95 (and macro-average F1 of 0.92) and an F1-score of 0.95 for patient voice only.

**Conclusion:**

The main conclusion resulting from this work is that combining social media data from platforms with different characteristics for training a patient and professional voice classifier does not result in best possible performance. We showed that it is best to train separate models per data source (Reddit and Twitter) instead of a model using the combined training data from both sources. We also found that it is preferable to train separate models per domain (cardiovascular and skin) while showing that the difference to the combined model is only minor (0.01 accuracy). Our highest overall F1-score (0.95) obtained for classifying posts as patient voice is a very good starting point for further analysis of social media data reflecting the experience of patients.

**Supplementary Information:**

The online version contains supplementary material available at 10.1186/s12911-021-01577-9.

## Background

### Introduction and motivation

There is a clear drive towards precision medicine in healthcare, to personalise a medicine treatment regimen for a particular patient, to ensure patients’ access to the right medicines in the right treatment pathway and to determine the right dosing amounts and/or dosing schedules at the right time. The better a treatment can be personalised, the more effective it will be for that patient. This is difficult to achieve in practice, however, a better understanding of how existing medicines and treatment regimens are being experienced by patients will help to personalise their medicine. Such personalisation may typically include interventions to enable an individual to feel better and more in control as their disease state progresses from diagnosis to disease management. In this paper, we focus on patients’ accounts related to different medications and medical conditions in social media and present work on classifying such data automatically using neural machine learning.

Research on analysing social media for health conditions or population health monitoring has increased considerably in recent years with the growing availability of data, Application Programming Interfaces (APIs) to collect it and the development of artificial intelligence (AI) algorithms to analyse it. As a result much work has focused on entity or concept tagging in social media posts, sentiment analysis or topic modelling of such data, and in the context of healthcare often with respect to particular domains which tend to be medical conditions or diseases. However, social media is made up of a mixture of a huge variety of information. For patient-centred healthcare analytics, it is therefore important to differentiate between posts which describe patients’ experiences and other types of posts.

The overarching goal of our research and development project is to perform data analytics of medical information in social media posts using Natural Language Processing (NLP). This requires entity and concept annotation of posts voicing patients’ experience as opposed to ones expressing professional experience, news and other types of content. In order to analyse social media in the context of precision medicine we must therefore identify those posts which represent the voice of the patient. We therefore treat this task as document-level classification task.

As we will explore in more detail in the following Related Work section, previous work in this area has focused on identifying personal experience posts limited to one social media platform (Twitter) and a dataset mentioning a set of medicines used for different medical conditions [1–4]. In our paper we extend the research in this area in three ways:

- we classify social media post in a three-way classification task by patient voice, professional voice and other posts,
- we extend the analysis done in previous work to include two data sources, Twitter and Reddit, and
- we examine if there are differences in the way patients and professionals post about different medical conditions by investigating model performances for two different domains (cardiovascular and skin diseases).

### Related work

The use of AI in healthcare is attracting enormous amounts of funding and investment both in research and industry, which has been accelerated dramatically during the COVID-19 pandemic. Davenport and Kalakota (2019) examined the potential for AI in healthcare in general and concluded that machine learning is fundamental in the development of precision medicine [5]. They state that AI algorithms will be applied increasingly within healthcare, with key applications being diagnosis and treatment recommendations, patient engagement and adherence, and administrative activities. The authors reflect on patient engagement and adherence being “the final barrier between ineffective and good health outcomes” and that this and other factors are increasingly being addressed by big data analysis efforts using AI. The paper states that relevant, targeted content provided to patients present itself a promising field of research in this area. We believe that the analysis of social media data related to medical conditions, medicines and side effects also has a role to play as part of the endeavour for achieving precision medicine.

#### Social media analysis for HealthCare

Antheunis et al. (2013) analysed patients’ and health professionals’ use of social media and found that patients primarily use Twitter for increasing their knowledge about a condition and exchanging advice, as opposed to Facebook which was used primarily by patients for social support and exchanging advice [6]. Their paper provides a review of the literature on this topic up until 2013 and sets out four motives for the use of social media and the internet more broadly in the context of health. These areas largely remain the same today, including searching information, providing social support, improving efficiency in terms of cost and quality of care and improving the relationship between patients and healthcare professionals. The authors’ analysis led them to conclude that patients’ main barrier for using social media was their concern for privacy and unreliability of information, as opposed to the professionals whose main barrier was inefficiency and lack of skills. Both types of users were expecting to use social media in the future which demonstrates its potential for data analytics.

Denecke et al. (2015) examined ethical issues related to the use of social media in the context of patient-centred care and found that the main issues in the use of social media in healthcare applications are the preservation of confidentiality and privacy [7]. The authors state that, while the availability of data can be beneficial, the abuse of data needs to be prevented.

In the context of cardiovascular diseases, one of the domains covered in our paper, Sinnenberg et al. (2016) carried out a large-scale Twitter analysis which focused on five cardiovascular diseases (hypertension, diabetes, myocardial infarction, heart failure, and cardiac arrest) using a number of related search queries. They collected tweets over a 5.5-year period, between 2009 and 2015 [8]. They excluded tweets that were automatically classified to be non-English and as well as any non-US tweets. They determined tweet location based on tweet coordinates (if available) or based on automatic mapping of locations mentioned in the tweets. They manually annotated a subset of 2500 tweets for frequency analysis with respect to the different cardiovascular disease types. They concluded that Twitter is a promising resource for the study of communication about cardiovascular diseases which is one of the reason we chose this domain for our own research. One major drawback of this study is that it does not differentiate between patients’ first hand experience of the disease and other types of posts. This is a gap that our paper tries to address.

Staying within this domain, Mandrola and Futyma (2020) provided a motivation and an overview of existing work on the analysis of social media data in the context of cardiology which is still fairly limited up to now [9]. They cite Sinnenberg et al.’s work [8] as well as another large-scale study which compared Twitter concordances mentioning adverse events with spontaneous adverse events reported to the Food and Drug Administration (FDA) [10] and found a high correlation between them. Mandrola and Futyma’s overview concludes that digital media brings change to healthcare and focuses on the positive aspects of what this might enable in the future.

Lu et al. (2020) reported on a study on temporal trends on mentions of and sentiment towards the flavour of e-cigarettes in social media data collected from Twitter [11]. Their study deliberately excluded Reddit posts as the authors expected sentiment analysis on Reddit posts to be harder as they are longer and provide more context. In contrast, we look at both Twitter and Reddit data to investigate how document classification models perform when tested in- and out-of data source to see how data source and size of context affect model performance.

Kim at al. (2020) presented experiments on binary classification of tweets mentioning methylphenidate or related brand names as either non-medical use or side effects using a Support Vector Machine (SVM) as their underlying machine learning algorithm [12]. Their best model, which was trained using a combination of training labels, features extracted from the tweet text as well as sentiment derived from each tweet, achieves high precision (>0.92) but fairly low recall.

In the context of skin diseases, another domain selected for our experiments, Okon et al. (2020) analysed a corpus of Reddit posts to evaluate dermatology patient experiences and therapeutics. They used a combination of topic modelling using Latent Dirichlet Allocation (LDA) [13], spectral clustering [14] and word cloud visualisations to identify cohesive themes within the topics emerging from the Reddit data but did not differentiate by patient experience or voice [15].

Finally, Meeking (2020) conducted a thematic analysis of patient experience tweets containing the keyword “radiotherapy”. Their analysis used a data set sampled across one year which was first manually screened for patient, healthcare professional, healthcare organisation by means of information provided either in the user profile on in the tweet text [16]. Our study attempts to automate this laborious manual screening step.

#### Personal experience posts

Jiang et al. (2016) understood the significance of distinguishing between social media posts reflecting the personal experience of posters and other types of posts [1]. They created a Twitter data set containing tweets related to four dietary supplements annotated as Personal Experience Tweet (PET) or non-PET. This corpus was created semi-automatically by bootstrapping tweets iteratively using a machine learning classifiers trained on different text and metadata-related features. They use this method for pre-annotation to speed up the manual annotation process. Their final annotated corpus contains 8770 tweets (2067 PET and 6703 non-PET). Inter-annotator agreement (IAA) was calculated using two annotators and achieved a Kappa score of 0.62 and an average agreement of 0.85% for both label types, PET and non-PET. Given that there is some distance between those scores and perfect agreement, the authors concluded that this kind of annotation has a level of subjectivity.

In a separate study, Sewalk et al. (2018) trained a patient experience classifier on tweets using SVM to train their models [17]. They report fairly low classifier precision (0.70), recall (0.69) and accuracy (0.83) as well as a fairly low IAA accuracy (0.69) when comparing pairs of Amazon Mechanical Turkers who were employed to label the collected tweets. Their low classifier performance is not unexpected given their low IAA.

Most recent work by the same group published by Zhu et al. (2020) compared previously tested Long Short-Term Memory (LSTM) and word embedding models [2, 3] to RoBERTa models [18], pre-trained, updated and trained from scratch for binary classification of PETs [4]. All RoBERTa models outperformed the baseline models significantly and updated pre-trained models performed best (F1-score=0.75). Their experiments and results are based on a publicly available Twitter dataset containing 12,331 tweets (2962 PET tweets and 9369 PET tweets) [2]. This dataset is a subset of tweets collected in 2015/16 mentioning 103 different medicines and was created using the same iterative approach as taken by Jiang et al. (2016) but this time a further annotator was used to adjudicate any doubly annotated tweets with disagreements in the labelling.

Motivated by this previous work and social media analysis in the context of medicine more generally, we present experiments for both Reddit and Twitter data and employ three-way document classification to identify posts that signify patient voice, professional voice, or other types of posts. We also present in- and cross-data-source and cross-domain classification performance of a trained Convolutional Neural Network (CNN) classifier. In the next section, we describe the data that was used and manually annotated for this purpose and provide detailed IAA scores for three annotators for a sizeable sub-part of the data to gain a better understanding of the difficulty and subjectivity of this task.

### Data

For the experiments described in this paper, we automatically collected social media posts from Twitter and Reddit reporting on either cardiovascular or skin conditions.

#### Data collection and preparation

Reddit posts were collected using the Pushshift Reddit API^1^ (to perform historical searches of posts) and the official Reddit API^2^ (to download the post content). We gathered Reddit posts by searching relevant subreddits for a set of manually collected search terms for skin and cardiovascular related conditions (see Supplementary Material for a full list of subreddits and search terms per domain). We used the same set of search terms to collect tweets from Twitter relevant to each domain.^3^ While we did not formally evaluate the relevance of each post to the two domains, previous research has showed that hand-selected search terms and hashtags lead to high recall and precision in that regard [19].

The data was then further filtered by removing duplicates (where a duplicate is defined as a post with an identical identifier or an identical text body to one already collected). The Reddit API still returns posts that are retroactively deleted by users, replaying the post text with “[deleted]”. These posts were also filtered out.

**Table 1:** Number of posts tokens per domain (cardiovascular and skin), data source (Reddit and Twitter) and overall counts

Domain$\backslash$data source	Reddit	Twitter	Total
Cardiovascular	8346	5622	13,968
Skin	11,331	4096	15,427
Both domains	19,677	9718	29,395

**Table 2:** Number of posts per domain, data source and label type for train (80%), test (20%) and overall

Domain	Data source	Patient voice	Professional voice	Other
Train (80%)				
Cardiovascular	Twitter	51	141	4307
	Reddit	2665	124	3889
Skin	Twitter	1264	179	1835
	Reddit	5604	46	3416
Test (20%)				
Cardiovascular	Twitter	13	35	1075
	Reddit	666	30	972
Skin	Twitter	316	45	457
	Reddit	1400	11	854
All				
Cardiovascular	Twitter	64	176	5382
	Reddit	3331	154	4861
Skin	Twitter	1580	224	2292
	Reddit	7004	57	4270

In total, we collected 29,383 posts, 19,669 Reddit posts and 9714 tweets (see Table 1 for individual counts per data source and domain).

#### Manual annotation

The manual annotation of the data was conducted using Doccano,^4^ an open source tool which supports collaborative annotation. The collected posts were loaded into Doccano prior to any pre-processing and were then annotated by a group of annotators trained in the annotation for this project using a set of detailed annotation guidelines. These guidelines were developed during an earlier round of annotation on data related to COVID-19 and further adapted when moving to the two domains presented in this paper (cardiovascular and skin conditions). The annotators labelled each post on the document label by post types but also marked up a set of entities (such as symptoms, medicines, feelings etc.) within posts. This paper does not report on the textual annotation of the data but focuses only on the document-level annotation and classification, and at the document level annotators were able to choose between the following six labels:

1. Patient voice: a post describing the first hand experience of a patient.
2. Professional voice: a post containing instructions or advice written by a medical healthcare professional, scientist or researcher (either uttered by the medical professional/scientist/researcher themselves or stated by someone else quoting them). This includes references to journal articles or posts with links by healthcare-related organisations and is not first hand patient experience. In some cases the link address is used to differentiate between professional voice and news.
3. News: a post written by a news professional, i.e. a journalist, news outlet, blogger or influencer, and is not a first hand experience. Direct references and links to news are labelled as such. Other posts containing links to news but with additional information by the poster are tagged depending on what the additional information contains.
4. Retweet: a post which is a retweet of a tweet (for data from Twitter only).
5. Not English: a post written in a different language, even if the keywords match.
6. Not relevant: a post which is either not related to the domain (cardiovascular or skin) or, if it is related to the domain, does not fit into any of the other categories.

The following are two example posts labelled with patient or professional voice:

- Patient voice post: *I was diagnosed with Atrial Fibrillation 5 years ago.*
- Professional voice post: *I am a cardiologist. In my professional opinion your cholesterol is pretty high. You should consider making some lifestyle changes.*

Patient voice clearly represents first hand patient experience whereas Professional voice captures the voice of a medical profession, scientist or researcher.

Annotators were instructed to assign exactly one label to each post with the exception of retweets in which case they are asked to annotate which other category the retweet belongs to. For the experiments reported in this paper, retweets are filtered out to avoid duplicate information and posted labelled as news, not English and not relevant are all grouped into one Other category. This means that in our experiments each post has only one of three labels: Patient voice, Professional voice or Other.

Table 2 lists overall counts for each type of label annotated in our data, per domain and data source as well as the distribution of label counts across the training data (80%) which we use for training our models and the test data (20%) used for evaluation.

**Table 3:** Number of tokens/unique tokens per data set and split

	Reddit	Twitter	Both data sources
Cardiovascular			
Train	831,169/26,037	119,087/16,118	950,256/34,998
Test	211,486/13,302	30,257/6729	241,743/17,094
Skin			
Train	1,159,225/29,176	98,410/13,639	1,257,635/35,854
Test	290,227/14,201	24,337/5483	314,564/16,731
Both domains			
Train	1,990,394/43,390	217,497/25,441	2,207,891/57,118
Test	501,713/21,779	54,594/10,201	556,307/26,444

**Table 4:** Inter-annotator agreement scores per domain and data source reported in terms of average per label F1 scores, macro-averaged F1 and accuracy (and standard deviation in brackets)

	Cardio/Reddit	Cardio/Twitter	Skin/Reddit	Skin/Twitter	All
F1: Other	0.90 (0.01)	0.93 (0.03)	0.89 (0.02)	0.95 (0.03)	0.93 (0.03)
F1: Patient voice	0.96 (0.01)	0.69 (0.09)	0.97 (0.01)	0.53 (0.19)	0.93 (0.03)
F1: Professional Voice	0.85 (0.03)	0.59 (0.07)	0.18 (0.15)	– (–)	0.59 (0.06)
Macro averaged F1	0.90 (0.03)	0.73 (0.06)	0.68 (0.05)	0.74 (0.11)	0.81 (0.04)
Accuracy (%)	0.94 (0.01)	0.87 (0.04)	0.95 (0.01)	0.91 (0.05)	0.92 (0.03)

Table 3 shows a breakdown of number of tokens versus unique tokens per data source, domain and overall for each data split. The biggest difference is in the number of tokens when comparing data sources (Reddit versus Twitter). Leaving aside the fact that we used approximately double the number of Reddit posts, they contain a lot more tokens compared to Twitter posts. This is due to the fact that Reddit posts tend to be much longer than tweets.

## Results

### Inter-annotator Agreement

We computed inter-annotator agreement (IAA) for the label assigned to each post to understand the difficulty of the classification task and to determine an upper bound for the performance that an automatic classifier could realistically obtain if it is trying to model human performance. We asked three expert annotators to label a total of 4000 randomly selected posts each (1000 per domain, cardiovascular and skin, and per data source, Reddit and Twitter).

We then calculated IAA for each of the three annotator pairs in terms of overall labelling accuracy, as well as precision, recall and F1-score for each label type, the same metrics we use for reporting system performance in our experiments described in the next section. This is done by essentially treating the mark-up of one annotator as the gold standard and another as system and by comparing the annotations of each of the three annotator pairs. We then computed averaged accuracy and F1-scores (per label as well as macro averaged F1) across the pairs.

Table 4 shows that average IAA is relatively high for Patient voice and Other at 0.93 F1 each and much lower for Professional voice at 0.59 F1. Overall IAA accuracy is 0.92.

### Experiments

In this section we describe a series of experiments to classify social media posts from Reddit and Twitter by the type of their voice (Patient voice, Professional voice or Other). We report model performance when making use of all of the available training data as well as results when training models per data source and domain.

**Table 5:** Results for the baseline model trained on all of the training data when testing it on all of test

Train all/test all	Precision	Recall	F1	Support
Other	0.88	0.86	0.87	3358
Patient voice	0.82	0.87	0.85	2395
Professional voice	0.35	0.23	0.28	121
Macro averages	0.68	0.65	0.67	5874
Accuracy	0.85			

We report precision, recall and F1 scores per label and overall as macro averages and accuracy as well as the number of test examples (Support)

**Table 6:** Result for the model trained on all Reddit data and testing it on all of test

Train Reddit/test all	Precision	Recall	F1	Support
Other	0.94	0.85	0.89	3358
Patient voice	0.81	0.94	0.87	2395
Professional voice	0.71	0.31	0.43	121
Macro averages	0.82	0.70	0.73	5874
Accuracy	0.87			

We report precision, recall and F1 scores per label and overall as macro averages and accuracy as well as the number of test examples (Support)

#### Experiment 1: Training and testing on all data

In this first experiment, we present the result for training our classifier on all of the annotated training data listed in Table 2, from both domains and data sources combined, and testing on all of the test data. We consider this model to be our baseline. The results reported in Table 5 show that the classifier is able to achieve reasonably high F1-scores for posts labelled as Other (F1=0.87) and Patient voice (F1=0.85). For Professional voice, the performance is quite low at 0.23 F1 but that is likely due to the relatively small number of training examples (the % of posts with that label in the test data is the same as in the training data). Overall accuracy for this model reaches 0.85 which compares with an IAA of 0.92 accuracy as the upper bound of what we believe a classifier could achieve with human intelligence.

#### Experiment 2: Training by data source (Reddit versus Twitter)

We ran a second experiment to see how performance changes when training per data source. We trained two classifiers, one on all of the training data from Reddit and one on the Twitter training data and tested on the different test sets.

**Table 7:** Result for the model trained on all Twitter data and testing it on all of test

Train Twitter/test all	Precision	Recall	F1	Support
Other	0.77	0.92	0.84	3358
Patient voice	0.85	0.63	0.72	2395
Professional voice	0.78	0.57	0.66	121
Macro averages	0.80	0.71	0.74	5874
Accuracy	0.79			

We report precision, recall and F1 scores per label and overall as macro averages and accuracy as well as the number of test examples (Support)

**Table 8:** Result for the Reddit and Twitter models on in- and out-of-source test data sets compared to the baseline model trained on all of the data

Model(s)	Other: F1	Patient voice: F1	Prof. Voice: F1	Macro F1	Acc.	Test
Reddit	0.94	0.95	0.86	0.92	0.95	Reddit:
Twitter	0.74	0.69	0.00	0.47	0.71	3933
All	0.85	0.88	0.30	0.68	0.86	
Reddit	0.83	0.50	0.00	0.44	0.73	Twitter:
Twitter	0.98	0.90	0.90	0.93	0.96	1941
All	0.90	0.64	0.26	0.60	0.83	
Reddit&Twitter	0.96	0.95	0.88	0.92	0.95	All:
All	0.87	0.85	0.28	0.66	0.85	5474

We also include the results for both models when tested each on in-source test data combined compared to the baseline model trained on all the data (last two rows). We report F1 scores per label, macro-average F1 and accuracy across all three label types as well as the size of the test set

**Table 9:** Result for the cardiovascular and skin-specific models on in- and out-of-domain test data sets compared to the model trained on all of the data

Model	Other: F1	Patient voice: F1	Prof. Voice: F1	Macro F1	Acc.	Test
Cardio	0.94	0.87	0.37	0.73	0.91	Cardio:
Skin	0.88	0.73	0.06	0.56	0.83	2791
All	0.93	0.86	0.21	0.67	0.90	
Cardio	0.69	0.63	0.07	0.46	0.66	Skin:
Skin	0.71	0.82	0.34	0.62	0.77	3083
All	0.73	0.83	0.16	0.57	0.76	

We report F1 scores per label, macro-average F1 and accuracy across all three labels as well as the size of the test set

The results in Tables 6 and 7 show that the model trained on the Reddit data performs a lot better overall (0.87 acc.) than the equivalent Twitter model (0.79 acc.) when tested on all of our test data and even outperforms the model trained on all of the data (see Experiment 1). This is in line with the IAA scores which are higher overall for Reddit than for Twitter and demonstrates that more consistently annotated data helps to improve classification performance.

The Twitter model performs better only on the Professional voice label (0.66 acc.). We believe this to be the result of it having access to almost double the number of training examples, 320 versus 170 post labelled Professional voice in the Reddit training data, and the test data containing a similar ratio of Twitter versus Reddit professional voice post, 75 versus 41 respectively.

When testing the Reddit and Twitter models on in- and out-of-source test data only (see Table 8) we found that models perform better on the data from the same source they were trained on. Their performance drops considerably (by >0.23 acc.) on out-of-source data. For comparison, the model trained on all the data (from both sources) performs roughly in the middle for each source-specific test sets. This is not unexpected as posts from Reddit and Twitter differ considerably in size of posts and therefore also their content and language. This means that we when building models for this social media classification task, it is important to stick with the same data source at train and run time. Adding more training data from a different source is not guaranteed to help to improve performance.

Table 8 also shows how the two data source models perform when combined, with each model tested only on its in-source test data. The overall performance of this combination on all of test is 10% higher in accuracy (0.95% acc.) than the baseline model which is trained on all of the available training data.

#### Experiment 3: Training by domain (cardio versus skin)

Finally, we performed an experiment looking at domain specific models. We trained two models, one only on posts related to cardiovascular disease and one only on skin disease related posts. We tested them on in- and out-of-domain test sets (see Table 9). The cardiovascular model performs with 0.08 higher accuracy on the cardiovascular test data than the skin model does. Similarly, the skin model performs with an accuracy of 0.11 higher on the skin test data than the cardiovascular model. We can conclude that in-domain knowledge helps to improve performance but, at least in this case, model performance does not suffer as much across domain compared to across source. Each domain-specific model only slightly outperforms the full model (see “All” in Table 9) trained on all of the data (cardiovascular and skin posts) in overall accuracy by 0.01 when tested on each domain-specific test set. This is mostly down to increased scores for the professional voice posts which are however not very frequent in the data.

### Methods

#### Algorithm

We used an off-the-shelf document classifier model architecture provided by spaCy (https://spacy.io) to perform multi-class document classification for our specific task to identify patient and professional voice posts in Reddit and Twitter data. Specifically, we used version 2.3.2 of the spaCy Python code library. Within this library, we used their TextCategorizer.^5^

The spaCy library does not provide many ways to configure the TextCategorizer but we document the two configuration parameters, and what we set these to, below for reproducibility:

- exclusive_classes—Set to “true” to make the model assume classes are mutually exclusive and “false” if an input document can be multiple classes. We set this to “true”.
- architecture—Pre-configured spaCy model architecture to use. We set this to “ensemble”.

The spaCy documentation describes the “ensemble” architecture as a stacked ensemble of a bag-of-words model and a neural network model.^6^ The CNN, a neural network architecture representing tokens in the document as vectors [20], has been mostly used for image analysis but in the last decade has been applied for different NLP tasks [21]. In spaCy, the CNN is used with mean pooling and attention. The “ngram_size” and “attr” arguments can be used to configure the feature extraction for the bag-of-words model. We used the default “ngram_size” and “attr” parameter values, which are set to 1 and “lower” respectively. This means the bag-of-words model used unigrams produced from tokenised text that was converted to all lowercase characters. So case differences did not result in distinct unigrams for the same word.

We recognise that more complex models could be employed, but spaCy’s TextCategorizer offers a strong baseline combined with a high level of convenience and efficiency in training and deploying classifiers. We did not perform parameter tuning given the limitation in parameters that are configurable in the library but also because we wanted to see how a text classifier as provided by spaCy performs out-of-the-box without tuning when trained using different types of training data sets. This is in line with Andrew Ng recent idea of encouraging the machine learning community to be more data-centric.^7^

#### Data preparation

We randomly split the annotated data into two subsets: train (80%) and test (20%). For this, we first shuffled the data, setting the random seed at 0 to ensure replicability. When splitting the data, we treated each tweet and Reddit post as a single document for the classifier and also ensured that the label distribution between train and test is the same (see Table 2).

We trained the TextCategorizer on the training data and evaluated it on the test data (see Experiment 1) and also experimented with training and testing models per data source and domain (see Experiments 2 and 3). The classifier’s training script accepts a list of selected class labels as a parameter, e.g. “Patient voice, Professional voice, Other”. While we kept Patient voice and Professional voice labels distinct for training the classifier, we combined all the other labels under the Other class. This greatly simplifies the multi-class classification task.

The TextCategorizer takes raw input text, tokenises it and removes stop words. We used the default tokenisation settings for English as defined in version 2.3.2 of the spaCy Python library.^8^

#### Evaluation metrics

We report inter-annotator agreement and document classification performance using standard metrics, including precision, recall and F1 scores for each label type. We also report macro-average F1 across all label types, which treats all label types equally in the evaluation, as well as accuracy which equates to micro-average precision, recall and F1. Both micro and macro-average metrics are useful for different reasons.

## Discussion

We found that overall IAA accuracy for our three-way classification task is fairly high at 0.92%. When examining the IAA scores more closely (Table 4), IAA is also high across the table for Other and Patient voice posts from Reddit. Due to the large number of annotations of posts for each of these subsets, we assume their IAA scores to be representative. When comparing their IAA scores across the two domains (cardiovascular and skin), it appears that average F1 scores for Other or Patient voice posts do not differ by a lot. This leads us to conclude that human annotators are able to classify Reddit posts on either domain as Patient voice reasonably consistently.

However, IAA is lower for Patient voice annotations of tweets (0.69 for cardiovascular disease related tweets and 0.53 for tweets on skin diseases). There are less than 50 Patient voice annotations and either no or less than 100 Professional voice annotations in the tweets sampled for computing IAA, those labelled as Other significantly outweigh the rest. For Professional voice, average F1 is 0.85 for less than 20 cardiovascular Reddit posts. For the other data subsets per domain and data source IAA is a lot lower. Therefore Patient voice IAA scores for tweets, in particular, and all Professional voice IAA scores listed in Table 4 should be treated with care and not assumed to be realistic estimates of IAA. More annotation examples are needed to get a better understanding of how well annotators agree on labelling them. With this caveat in mind, it does still appear that IAA is lower on tweets than on Reddit posts. We believe the reason for this to be the fact that tweets are much shorter and it is more difficult to label them manually due to the limited context they provide for this classification task.

With respect to the three experiments (see Tables 5, 6, 7, 8, 9) conducted with different variations of training and test datasets (overall, by data source and by domain) we found the best performing models to be those which are trained on separate Reddit and Twitter posts. This result was not unexpected as they encompass clear differences, most of all size of posts and therefore level of detail in the language used. However, in machine learning there is a tendency to train models with as much data one can get access to and so our results show that throwing all our available data at this particular problem is not the right approach.

When training by medical domain, however, our results show that, in the case of cardiovascular and skin diseases, training by domain as opposed to training a combined model does not lead to considerably different results. Each domain-specific model is trained on much less data than the combined model and still achieves a slightly higher accuracy (0.91 for cardiovascular and 0.77 for skin). On the other hand, the model trained on data from both domains also does not harm classification performance in the same way as the model combining data from two data sources. We suspect the reason for this is that patients and medical professionals use similar language when discussing medical conditions and diseases. While the medical terminology itself differs across domains, the context in which it appears provides sufficient overlapping signals and clues for the model learned from the combined training data to classify posts almost as accurately as the domain-specific models.

## Conclusions

In this paper we presented a series of experiments on classifying social media data collected from Reddit and Twitter related to two different health conditions by patient and professional voice. We described the data used for training document classification models and how it was annotated, as well as presented average inter-annotator agreement scores three sets of double-annotations. We showed that this classification task can be done relatively consistently manually (with an overall IAA accuracy of 0.92), that annotators agree roughly equally on this task for each domain but that they agree more when annotating Reddit posts compared to Twitter posts.

We have presented a number of experiments using all of our annotated training and test data or sub-sets for training models by source and domain and have tested in- as well as out-of-source or domain. Based on the results we have learned that for the classification task to differentiate between patient voice, professional voice and other posts:

- it is best to train separate models per data source (Reddit and Twitter) instead of a model using the combined training data from both sources.
- it is better to train separate models with data coming from different domains (cardiovascular and skin) but their improvement over the combined model is marginal.

Training models by data source and testing on in-source data has achieved high accuracy scores (>0.95 accuracy). We note that the Twitter model is trained on approximately half the number of posts than the Reddit model, and its training data is a lot smaller in terms of number of overall word tokens. Nevertheless, both perform equally well overall. However, when tested out-of-source, each model’s performance drops drastically. This means that to maximise accuracy and F1 scores these two models should be ideally used separately for classifying data from their own source. Using them in this way across the entire test set, each model run only on in-source test posts, we achieved an overall best combined performance for classifying patient voice (F1=0.95), professional voice (F1=0.88) and other posts (F1=0.96) with an overall accuracy of 0.95 and a macro-average F1 of 0.92. Direct comparison with previous work by other research groups in this area is not possible due to the use of different data sets and variation in the framing of the task.

We also found that adding more training data from a different domain does not improve performance of domain-specific models, but also does not seriously harm overall accuracy. This suggests that there must be some similarities in the language used in the context of patient and professional voice posts written for different medical conditions, even if the condition- or medicine-specific terms differ for each domain.

Our best performing classifier combination reaches a decent performance for identifying patient voice. Being able to differentiate accurately between patient voice and other health related posts is a vital first step for healthcare analytics on social media. Being able to do this well with an off-the-shelf classification tool without any parameter optimisation is an added bonus.

## Supplementary Information

**Additional file 1.** File contains the list of subreddits and search terms used for collecting the data for each domain.

## Data Availability

We provide the list of subreddits and search terms, which we used to collect the data for this research and development project, in the Appendix. The annotation labels and examples are also described in this paper. The third-party tools (classifier and annotation tool) used for this work are freely available and details on the classifier set-up and model parameters are provided in this paper. For more information about this project and the data please contact Elizabeth A.L. Fairley.

## Abbreviations

API: Application programming interface
CNN: Convolutional neural network
FDA: Food and Drug Administration
IAA: Inter-Annotator Agreement
LSTM: Long Short-Term Memory
NLP: Natural language processing
PET: Personal Experience Tweet
SVM: Support vector machine

## Footnotes

1 https://github.com/pushshift/api.

2 https://www.reddit.com/dev/api/.

3 The data was gathered over the time period of 2017-01-01 to 2020-07-17.

4 https://github.com/doccano/doccano.

5 https://spacy.io/api/textcategorize.

6 https://spacy.io/api/architectures.

7 https://analyticsindiamag.com/big-data-to-good-data-andrew-ng-urges-ml-community-to-be-more-data-centric-and-less-model-centric/.

8 https://github.com/explosion/spaCy/tree/v2.3.2/spacy/lang/en.
