# Supplementary material for: Classifying patient and professional voice in social media health posts
Source: BMC Med Inform Decis Mak. 2021 Aug 18;21:244. doi: 10.1186/s12911-021-01577-9 (PMC8371035; doi:10.1186/s12911-021-01577-9)
Supplement: Supplementary file 1 — Additional file 1. File contains the list of subreddits and search terms used for collecting the data for each domain. [file 12911_2021_1577_MOESM1_ESM.docx]

**Supplementary Material**

The following subreddits were searched for each domain:

Cardiovascular: askCardiology, Cardiomyopathy, CHDs, Cholesterol, HeartAttack, HeartDisease, Heartfailure, hypertension, ihadastroke, PulmonaryHypertension and stroke

Skin: Accutane, AusSkincare, Dermatology, Eczema, EczemaCures, EczemaDiet, popping, Psoriasis, skin, SkincareAddicts, SkincareAddition and teenagers

The following search terms were used to search Reddit and Twitter per domain:

Cardiovascular Reddit: Accupril, ACE inhibitor, ACEi, Actilyse, afib, afib treatment, Altace, angina, apixaban, ARB, ARBs, arrhythmia, asystole, atherosclerosis, atorvastatin, atrial, atrial fibrillation, atrial flutter, Benicar, Beta blocker, Beta blockers, Blood clot, blood clots, blood pressure symptoms, Blood thinner, Blood thinners, Blopress, bradycardia, Calcium channel blocker, Calcium channel blockers, cardiac arrest, cardiac failure, cardiac infarction, cardiomyopathy, cardiovascular, chf, cholesterol, congenital, Coversyl, Cozaar, Crestor, dabigatran, Diovan, Diuretic, Diuretics, DOAC, DOACs, edoxaban, Eliquis, Entresto, fast heart rate, fibrillation, heart, heart arrest, heart attack, heart disease, heart failure, heart rate, high blood pressure symptoms, high blood presure, high heart rate, high pulse rate, high resting heart rate, htn, hypertension, irregular heartbeat, Lescol, Lipitor, Lipostat, Lixiana, losartan, macitentan, myocardial infarction, myocardial necrosis, Nilemdo, Nitrates, normal heart rate, Norvasc, Opsumit, palpitations, pericarditis, Pradaxa, Praluent, Privinil, resting heart rate, Retavase, rivaroxaban, rosuvastatin, selexipag, signs of high blood pressure, statins, stroke, systemic embolism, TNKase, transient ischaemic attack, Uptravi, valsartan, valve disease, Vasotec, ventricular fibrillation, vfib, Warfarin, Xarelto, Zestril, Zocor

Cardiovascular Twitter: Accupril, ACE inhibitor, ACEi, Actilyse,afib, afib treatment, Altace, angina, apixaban, ARB, ARBs, arrhythmia, asystole, atherosclerosis, atorvastatin, atrial, atrial fibrillation, atrial flutter, Benicar, Beta blocker, Beta blockers, Blood clot, blood clots, blood pressure symptoms, Blood thinner, Blood thinners, Blopress, bradycardia, Calcium channel blocker, Calcium channel blockers, cardiac arrest, cardiac failure, cardiac infarction, cardiomyopathy, cardiovascular, chf, cholesterol, congenital, Coversyl, Cozaar, Crestor, dabigatran, Diovan, Diuretic, Diuretics, DOAC, DOACs, edoxaban, Eliquis, Entresto, fibrillation, heart arrest, heart disease, heart failure, high blood pressure symptoms, high blood presure, high pulse rate, htn, hypertension, irregular heartbeat, Lescol, Lipitor, Lipostat, Lixiana, losartan, macitentan, myocardial infarction, myocardial necrosis, Nilemdo, Nitrates, Norvasc, Opsumit, palpitations, pericarditis, Pradaxa, Praluent, Privinil, Retavase, rivaroxaban, rosuvastatin, selexipag, signs of high blood pressure, statins, stroke, systemic embolism, TNKase, transient ischaemic attack, Uptravi, valsartan, valve disease, Vasotec, ventricular fibrillation, vfib, Warfarin, Xarelto, Zestril, Zocor

Skin Reddit and Twitter: balneum, dermatitis, diprobase, E45, eczema, flakey skin, irritated skin, itchy skin, oilatum, patchy skin, psoriasis, skilarence, sore skin
